# Duration of Environmental Enrichment Determines Astrocyte Number and Cervical Lymph Node T Lymphocyte Proportions but Not the Microglial Number in Middle-Aged C57BL/6 Mice

**DOI:** 10.3389/fncel.2020.00057

**Published:** 2020-03-18

**Authors:** Gaurav Singhal, Julie Morgan, Magdalene C. Jawahar, Frances Corrigan, Emily J. Jaehne, Catherine Toben, Jim Manavis, Anthony J. Hannan, Bernhard T. Baune

**Affiliations:** ^1^Psychiatric Neuroscience Lab, Discipline of Psychiatry, The University of Adelaide, Adelaide, SA, Australia; ^2^Division of Health Sciences, The University of South Australia, Adelaide, SA, Australia; ^3^School of Psychology and Public Health, La Trobe University, Melbourne, VIC, Australia; ^4^Faculty of Health, Centre for Neurological Diseases, School of Medicine, The University of Adelaide, Adelaide, SA, Australia; ^5^Melbourne Brain Centre, Florey Institute of Neuroscience and Mental Health, The University of Melbourne, Melbourne, VIC, Australia; ^6^Department of Psychiatry, Melbourne Medical School, The University of Melbourne, Melbourne, VIC, Australia; ^7^Department of Psychiatry, University of Münster, Münster, Germany

**Keywords:** environmental enrichment, immune, brain, microglia, astrocytes, T cells

## Abstract

Environmental enrichment (EE) has been shown to modulate behavior and immunity. We recently reported that both short and long-term EE enhance baseline locomotion and alleviate depressive-like behavior, but only long-term EE affects locomotion adversely in a threatening environment and enhances anxiety-like behavior in middle-age mice. We have now investigated whether the observed changes in behavior after short- and long-term EE were associated with underlying immune changes. Hence, at the end of behavioral testing, mice were sacrificed, and brains and cervical lymph nodes were collected to investigate the differential effects of the duration of EE (short- and long-term) on the number of immunopositive glial cells in the dentate gyrus, CA1, CA2, and CA3 regions of the hippocampus and proportions of T cell subsets in the cervical lymph nodes using immunohistochemistry and flow cytometry, respectively. EE, regardless of duration, caused an increase in microglia number within the dentate gyrus, CA1 and CA3 hippocampal regions, but only long-term EE increased astrocytes number within the dentate gyrus and CA3 hippocampal regions. A significantly higher proportion of CD8^+^ naive T cells was observed after long-term EE vs. short-term EE. No significant differences were observed in the proportion of central memory and effector memory T cells or early activated CD25^+^ cells between any of the test groups. Our results suggest that EE, irrespective of duration, enhances the numbers of microglia, but long-term EE is required to modify astrocyte number and peripheral T cell proportions in middle-aged mice. Our findings provide new insights into the therapeutic effects of EE on various brain disorders, which may be at least partly mediated by glial and neuroimmune modulation.

## Introduction

Environmental enrichment in rodents has been studied extensively in the last decade for its beneficial effects on neuroimmune mechanisms that enhanced our understanding and approach in dealing with psychiatric disorders. EE promotes cognitive stimuli in the experimental animals with the use of running wheels, novel objects, puzzles (mazes, plastic tubes in different configurations) and accessories (toys, ropes, ladders, tunnels, hanging objects, house, ramps, and platforms; Singhal et al., 2014a). Brain functions including behavior, cognition, and locomotion, improve after EE (Soffié et al., 1999; Tees, 1999; Williams et al., 2001; Kempermann et al., 2002; Leggio et al., 2005; Bennett et al., 2006; Segovia et al., 2006; Leal-Galicia et al., 2008; Harati et al., 2011). EE has also shown beneficial effects in several rodent models of psychiatric and neurodegenerative disorders, such as Huntington's disease (Spires et al., 2004; Wood et al., 2010), Alzheimer's disease (AD) (Jankowsky et al., 2003, 2005), Parkinson's disease (Faherty et al., 2005), stroke (Buchhold et al., 2007), traumatic brain injury (Hamm et al., 1996; Passineau et al., 2001), multiple sclerosis (Pusic and Kraig, 2014), schizophrenia (Burrows et al., 2015), anxiety (Benaroya-Milshtein et al., 2004), and depression (Koh et al., 2007). Extensive evidence suggests that the functional behavioral changes in the brain post EE correlate with the underlying immune changes (Soffié et al., 1999; Cotter et al., 2001; Ehninger and Kempermann, 2003; Laviola et al., 2004, 2008; Nagele et al., 2004; Williamson et al., 2012; Beauquis et al., 2013). However, the effects of the duration of EE, if any, on neuroimmune functions are still unknown.

Studies have shown EE induces a significant alteration within rodent brains in molecular configuration and function of immune factors. In particular, glial cells, T cell subsets, and pro- and anti-inflammatory cytokines undergo a significant change in molecular configuration post EE. Brain glial cells are also known to modulate neurobiology through the expression of pro- or anti-inflammatory factors (Kuzumaki et al., 2010; Li et al., 2011; Singhal et al., 2014b; Von Bernhardi et al., 2015). However, the reported neuroimmune outcomes post EE varied greatly amongst studies, which may be accounted for by confounders such as strain, gender, and age, as well as duration and timing of EE. For instance, long-term EE from weaning to 23 months of age reduced the number and size of astrocytes in the hippocampus and corpus callosum in male Wistar-derived rats, which was in contrast to hypertrophied astrocytes and memory deficit of rats kept in standard housing conditions (Soffié et al., 1999). Likewise, EE significantly increased both astrocyte (GFAP^+^) and microglia (IBA1^+^) antigen expression within the dentate gyrus, but not in the CA1, CA3, or cortex of adult male rats that were housed for 12 h in an enriched environment (Williamson et al., 2012). In a separate study, juvenile Sprague-Dawley rats from stressed pregnancies showed an increase in the basal circulating levels of CD4^+^ and CD8^+^ T lymphocytes, increased levels of cytokine IL-2, and reduced levels of cytokine IL-1β when housed in short-term enriched conditions at an early age (Laviola et al., 2004).

EE for rodents in large cages with toys and other accessories has shown beneficial effects on brain glial cell morphology and numbers in models of several neurodegenerative and psychiatric disorders such as AD (Beauquis et al., 2013), schizophrenia (Rahati et al., 2016) and depression (Laviola et al., 2008). While abnormalities in brain glial cell morphology and functioning are the established etiologies of these psychiatric disorders (Cotter et al., 2001; Nagele et al., 2004), the effects of different EE protocols and inclusion or exclusion of EE objects on glial cells may differ in different brain regions. For example, while treatment with EE led to a significant increase in the number of new astrocytes in layer 1 of the motor cortex, physical exercise using voluntary wheel running, a technique which has also been used as a tool to enrich the environment of rodents extensively, induced the proliferation of microglia in superficial cortical layers of several brain regions (Ehninger and Kempermann, 2003). Interestingly, physical exercise is, in itself, a known inducer of neurogenesis (Van Praag et al., 2005; Chen et al., 2006). This evidence suggests that the inclusion of running wheels in EE protocols may create a bias and could be confounding to the results on the effect of EE alone on immune functions. However, it must be noted that EE animals, although not as active in the absence of running wheels, do explore EE tools in their cages, which adds to additional physical activity for them compared to control (standard-housed) animals.

Hence, in the absence of direct assessment, the role of the duration of the EE (without running wheels) in the modulation of neuroimmune mechanisms remains unclear. In our recently published work, we reported an increase in baseline locomotion and decrease in depressive-like behavior after both short and long-term EE in middle-aged mice. Furthermore, long-term EE affected locomotion adversely in a threatening environment and was found to be anxiogenic (Singhal et al., 2019). We, therefore, wanted to investigate if the observed changes in behavior in response to the different duration of EE were the result of underlying neuroimmune outcomes. Hence, all mice that underwent behavioral battery were sacrificed, and brains and cervical lymph nodes were collected for the analysis of change in numbers of astrocytes and microglia in the dentate gyrus, CA1, CA2, and CA3 regions of the hippocampus and T cell subset proportions from cervical lymph nodes. We believe the findings from this study will benefit future research targeting EE to enhance neural function through immunomodulation during middle age.

## Materials and Methods

This study is a continuation of the behavioral and gene expression study that we have published recently. Hence, animals and experimental design are similar to what we have already reported. The behavioral battery is discussed in detail in the published article (Singhal et al., 2019).

### Animals

Wild-type C57BL/6 mice (*n* = 58; 31 males and 27 females), parental substrain Nhsd (derived from the National Institutes of Health, Bethesda, Maryland, USA), were bred in-house in the laboratory animal services (LAS) facility at the University of Adelaide and housed in individually ventilated cages (IVCs) under controlled conditions of temperature (21 ± 1°C), humidity (55%) and a 12–12 h dark-light cycle. During the experimental timeline, the C57BL/6Nhsd mouse line was inbred between 9 and 13 generations. Mice were provided *ad libitum* standard laboratory food and water. C57BL/6 mice were selected for the study since they have been characterized well in terms of humoral and cellular neuroimmune responses to environmental factors (Song and Hwang, 2017).

Ethics approval for performing experiments on C57BL/6 mice was received from the University of Adelaide Animal Ethics Committee (M216-12), and all guidelines as prescribed for handling the experimental animals were followed during the study.

### Experimental Design

Once the desired age (12 weeks or 8 months of age) was reached, those mice without signs of injury or illness, hence not challenged immunologically, were randomly allocated into either treatment (short-term and long-term EE) or control groups (*n* = 12–16). Mice were randomly paired (males and females paired separately) and transferred to open top cages (2 mice per cage). Control mice received no treatment and were kept in cages with the following dimensions: 48.5 × 15.5 × 12 cm. EE mice were kept in plexiglass cages with dimensions: 37 × 20.5 × 13.5 cm, as these had more breadth and depth to provide extra space for the objects associated with EE.

**Figure 1:**
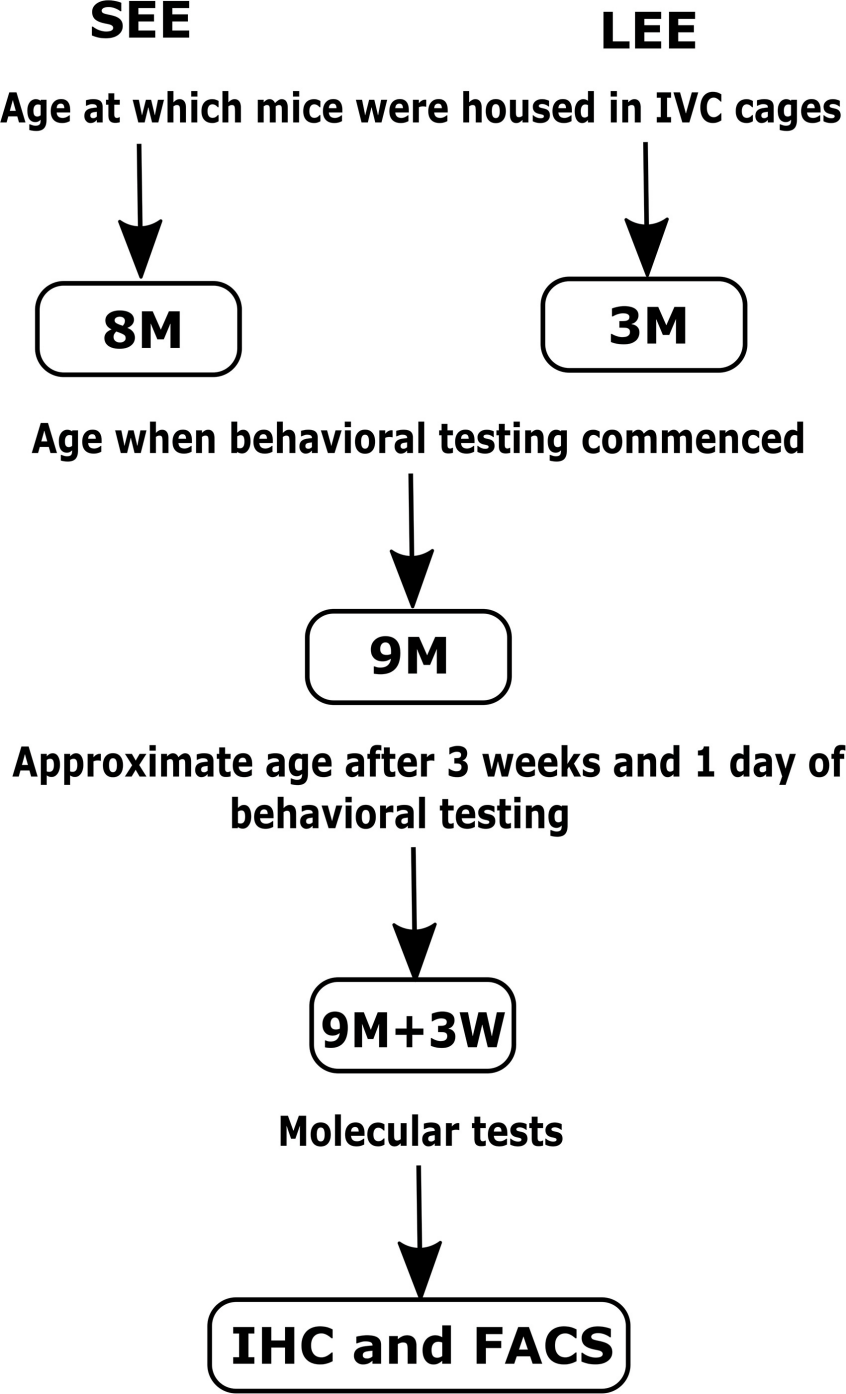
Schematic representation of the short-term (SEE) and long-term (LEE) EE protocols. M, months; W, weeks; IHC, Immunohistochemistry; FACS, Fluorescence-activated cell sorting.

The group assigned as short-term EE were provided with a variety of non-toxic novel objects (house, colored balls, toys, hanging toys, ladder, and tunnels) and extra bedding as per previously published protocols (Spires et al., 2004; Jankowsky et al., 2005; Leggio et al., 2005) for 4 weeks before behavioral testing, i.e., starting at 8 months of age and ending at 9 months of age. Four weeks of EE is considered equivalent to 1 month of EE for the sake of simplicity. The objects were changed once every week to maintain novelty (during the change of cages on Fridays, starting Friday of week 1, to minimize handling stress to mice). Similarly, the group assigned as long-term EE were provided with a variety of non-toxic novel objects and extra bedding for 6 months starting at 3 months of age, with objects changed once every week to maintain novelty. At the time of change of cages, mice were weighed on a digital weighing scale, the analyses of which have been published (Singhal et al., 2019). The objects remained in the cages throughout the 3 weeks of the behavioral testing period that followed the short-term and long-term treatments (Figure 1). Mice in both groups also received nesting material (paper shreds) during the experiments. Mice were monitored for dominancy throughout the experiments, and those found to be dominant were segregated to prevent dominance effects on neuroimmune mechanisms.

It is important to note that the behavioral testing ended with the forced swim test a day before tissue collection. For full behavioral testing protocol and data analyses, see the published paper (Singhal et al., 2019). We used both male and female mice in approximately equal numbers to represent findings from a population comprising of both sexes, however, it is important to note that we could not perform sex analysis due to a low sample size for both males and females when taken separately (Table 1).

**Table 1:** Mouse numbers for behavioral and molecular analysis in short- and long-term control and EE groups.

**Treatment**	**Behavioral analysis *n* (males: females)**	**FACS *n* (males: females)**	**IHC *n* (males: females)**
Short-term control	15 (9:6)	7 (4:3)	6 (3:3)
Short-term EE	12 (6:6)	6 (3:3)	6 (3:3)
Long-term control	16 (8:8)	7 (4:3)	6 (3:3)
Long-term EE	15 (8:7)	8 (4:4)	6 (3:3)

*FACS, fluorescence-activated cell sorting; IHC, immunohistochemistry*.

### Molecular Analysis

After completion of either short-term or long-term EE treatments and behavioral testing as has been reported (Singhal et al., 2019), mice were terminally anesthetized with a lethal intraperitoneal dose of pentobarbital (60 mg/kg IP). Animals utilized for immunohistochemistry were perfused via transcardiac injection with 10% neutral buffered formalin, with the brains rapidly removed and placed in 10% formalin until further procedures. Animals utilized for FACS had their draining cervical lymph nodes of the brain removed and collected in Roswell Park Memorial Institute (RPMI+) medium.

#### Immunohistochemistry

Brains preserved in 10% formalin were cut into five 3 mm coronal slices and following overnight treatment with increasing concentrations and durations of ethanol, xylene and paraffin baths; the sliced brain samples were embedded in paraffin wax. The hippocampus was then serially sectioned, with six sections 150 μm apart taken.

For immunohistochemistry, on day 1, sections were dewaxed and dehydrated in ethanol, and endogenous peroxidase activity was blocked by incubation with 0.5% hydrogen peroxide in methanol for 30 min. Antigen retrieval was performed by heating at close to boiling point for 10 min in citrate buffer, and slides were then allowed to cool below 40°C before further processing. The appropriate primary antibody (IBA1 for microglia, 1: 10000; GFAP for astrocytes 1: 40000; Abcam, United Kingdom) was applied to the slides which were then left to incubate overnight, allowing primary antibodies to bind to the target antigen. On day 2, the IgG biotinylated antibody of rabbit (same as primary antibodies) was added and allowed to react with primary antibodies for 30 min. The formed immune complex was then further amplified by incubating slides with a biotin-binding protein, streptavidin-peroxidase conjugate, for 60 min. The immune complex was then visualized with precipitation of DAB in the presence of hydrogen peroxide. Slides were washed to remove excess DAB and lightly counterstained with hematoxylin, dehydrated and mounted with DePex.

All slides were digitally scanned (Nanozoomer, Hamamatsu City, Japan) and then viewed with the associated software NDP view (version 1.2.2.5). Immunopositive cells in the dentate gyrus, CA1, CA2, and CA3 regions of the hippocampus were counted manually for statistical analysis. The hippocampus, in particular, the dentate gyrus region, has been shown to play a vital role in the regulation of behavior and memory in response to external stimulus and shows alterations after EE (Scoville and Milner, 1957; Schacter et al., 1996; Nilsson et al., 1999; Campbell and MacQueen, 2004; Kim et al., 2004; Bruel-Jungerman et al., 2005; Chen et al., 2006; Engin and Treit, 2007; Williamson et al., 2012; Sampedro-Piquero et al., 2013). Hence, we selected the hippocampus for the analysis of glial cells.

Freehand boxes were drawn to cover the entire dentate gyrus regions of the six stained sections followed by counting of the cells within the boxes. For each section, the count of cells was then divided with the area of the box (in mm^2^) to get the number of cells/mm^2^. The average of six sections represented the value for one mouse and was utilized during statistical analysis. It is important to note that unlike the dentate gyrus, the other regions of the hippocampus are not clearly demarcated. Hence, the sampling boxes technique was utilized to analyze the number of immunopositive glial cells in the CA1, CA2, and CA3 regions of the hippocampus. To achieve this objective, square boxes of equal area were drawn in the CA1, CA2, and CA3 regions of the hippocampus, and the cells were counted within boxes. For each section, the cell count was then divided with the area of the box (in mm^2^) to get the number of cells/mm^2^. The average of six sections represented the value for one mouse and was utilized during statistical analysis.

#### Peripheral T Cell Immunophenotyping Using Fluorescence-Activated Cell Sorting (FACS)

FACS was applied for the detection of T cell proportions and characterization of their phenotype in the cervical lymph nodes of mice. This included CD4^+^ and CD8^+^ T cell subpopulations (Naïve or T_N_, Central memory or T_CM_ and Effector memory or T_EM_), and early activated T cell phenotype (CD25^+^).

Cervical lymph nodes collected in RPMI+ were passed through a 0.1 μ sieve (BD) using additional RPMI+ and centrifuged to separate cells from tissue debris. Retrieved lymph node cells were counted on a hemocytometer and resuspended in PBS to a final concentration of 2 × 10^6^ cells/ml. 250 μL of the cell suspension was then washed once with FACS buffer (PBS with 1% heat-inactivated bovine serum albumin) and blocked with 10 μL 0.5 mg/mL Fc block. An eight-color staining panel was used to characterize the CD4^+^ and CD8^+^ T cells. Unstained cells were used to gate out autofluorescent cells while single stained and fluorescence minus one (FMO) stained cells were used to control for spectral overlap or distinguishing between negative and positive cells, respectively (non-specific binding). Cells were incubated for 30 min at room temperature with the respective mAbs (Table 2). Following this, the cells were washed twice before resuspension in 300 μL FACS buffer. Cells were analyzed using the Gallios flow cytometer, and 100,000 events were acquired. The data obtained were analyzed using FCS Express software (version 4). Forward side scatter gating on acquired data distinguished singlet from doublet cell populations from which CD45^+^ cells were gated. CD4^+^ or CD8^+^ T cell subpopulations were then gated on CD45^+^ CD3^+^ T cells. Percentages of CD4^+^ or CD8^+^ gated T cells were used to calculate total cell numbers when multiplied with cell counts. Further gating on CD44^+^ and CD62L^+^ cell populations in the FCS Express software-enabled identification of naïve and memory T cells subsets (T_N_, T_CM_, and T_EM_).

**Table 2:** Monoclonal antibodies used for T cell immunophenotyping.

**mAb**	**Clone**	**Fluorochromes**	**BD biosciences Cat. No**.	**Conc. (mg/mL)**	**Antigen distribution/function**
CD3	145-2C11	FITC	553061	1.0 × 10^3^	T-cell identification marker
CD45	30-F11	V500	561487	2.0 × 10^3^	Nucleated hematopoietic cell lineage marker; common leukocyte antigen
CD4	GK1.5	APC-H7	560181	2.0 × 10^4^	T helper cell co-receptor for MHC II-restricted antigen induced T-cell activation
CD8	53-6.7	PerCP-Cy5.5	551162	2.0 × 10^4^	Cytotoxic T-cell Co-receptor for MHC I restricted antigen induced T-cell activation
CD25	3C7	PE	561065	2.0 × 10^3^	Early T-cell activation marker
CD44	IM7	PerCy7	560569	5.0 × 10^4^	Activation marker for effector or memory T-cells; attachment and rolling
CD62L	MEL-14	V450	560507	5.0 × 10^5^	T-cell homing receptor; transmigration
CD69	H1.2F3	APC	560689	2.0 × 10^3^	Early T-cell activation marker

*mAb, monoclonal antibody; Conc., concentration; MHC, major histocompatibility complex*.

### Statistical Analysis

Statistical analyses were conducted using GraphPad Prism version 7.02 (GraphPad Software Inc.). All data outliers were removed using the ROUT method, and normality of data distribution was determined by visual inspection of histograms. Comparisons between the treatments (short-term and long-term EE) and respective controls were performed using a fixed-effect model of two-way ANOVA. The two-way ANOVA statistical model was chosen to elucidate a two-way interaction effect between the two independent variables (treatment and duration) on the dependent variable (count of glial or T cells). The multiple comparisons *post-hoc* Holm-Sidak's test was used to confirm significant interactions between groups. Results are presented as mean ± SEM. Differences are considered statistically significant when *p* < 0.05.

## Results

### Alteration in Brain Microglia and Astrocyte Activation Marker Number in C57BL/6 Mice Exposed to Short or Long-Term EE

Immunopositive microglia and astrocytes were counted in the dentate gyrus, CA1, CA2, and CA3 regions of the hippocampus and analyzed statistically using two-way ANOVA with *post-hoc* Holm-Sidak's multiple comparison test. Supplementary Figures 1–8 shows the representative immunohistochemical images of the number of IBA1^+^ microglia and GFAP+ astrocytes in the dentate gyrus, CA1, CA2, and CA3 regions of the hippocampus in short- and long-term control and EE groups.

#### Both Short- and Long-Term EE Induced a Significant Increase in the Number of IBA1^+^ Microglia in the Dentate Gyrus, CA1, and CA3 Regions of the Hippocampus

**Figure 2:**
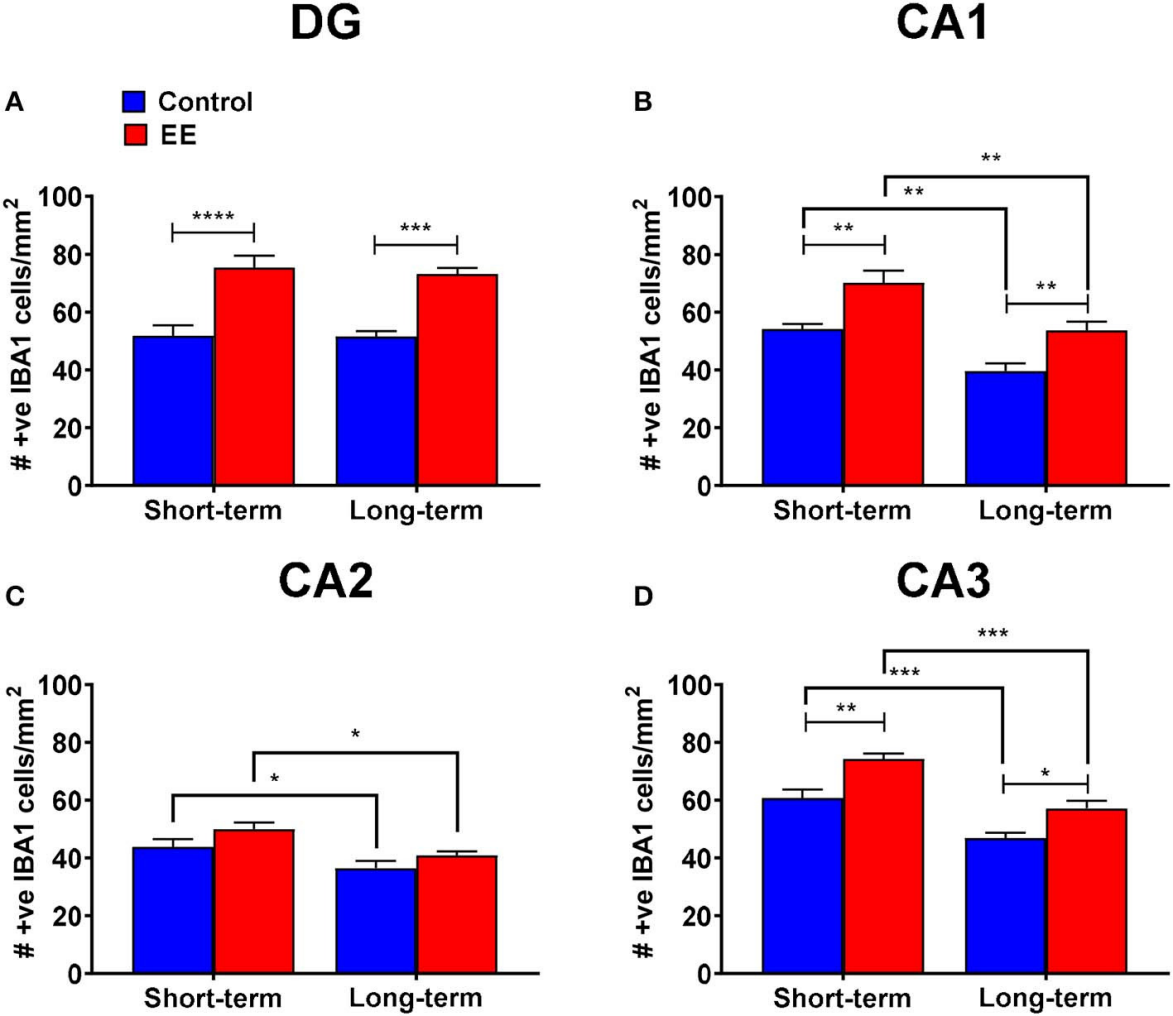
IBA1^+^ microglia in DG. **(A–D)** Number of IBA1^+^ microglia in the dentate gyrus (DG), CA1, CA2, and CA3 regions of the hippocampus, respectively. All data represented as mean ± SEM, *n* = 6 per group. *p < 0.05, **p < 0.01, ***p < 0.001, ****p < 0.0001.

A non-significant interaction effect (*F*_(1,20)_ = 0.09; *p* = 0.7615) and the main effects of duration (*F*_(1,20)_ = 0.15; *p* = 0.6994) were noted for IBA1^+^ microglia within the dentate gyrus. However, the main effect of treatment was significant (*F*_(1,20)_ = 53.1; *p* < 0.0001). Both short-term and long-term EE treated mice showed significantly higher IBA1^+^ cell numbers than their respective controls in the dentate gyrus (75.4 ± 4.2 vs. 51.7 ± 3.6; *p* < 0.0001 and 73.2 ± 2.1 vs. 51.5 ± 2.0; *p* = 0.0002, respectively; Figure 2A).

During the analysis of IBA1^+^ microglia in the hippocampal CA1 region, the interaction effect was non-significant (*F*_(1,20)_ = 0.09; *p* = 0.7594), however, the main effects of both duration (*F*_(1,20)_ = 25.17; *p* < 0.0001) and treatment (*F*_(1,20)_ = 23.61; *p* < 0.0001) were significant. Both short-term and long-term EE treated mice showed significantly higher IBA1^+^ cell numbers than their respective controls (70.1 ± 4.3 vs. 54.2 ± 1.8; *p* = 0.0031 and 53.7 ± 3.1 vs. 39.6 ± 2.7; *p* = 0.0087, respectively; Figure 2B). Similarly, both short-term control and EE mice showed significantly higher IBA1^+^ cell numbers than their respective long-term cohorts (54.2 ± 1.8 vs. 39.6 ± 2.7; *p* = 0.0034 and 70.1 ± 4.3 vs. 53.7 ± 3.1; *p* = 0.0024, respectively; Figure 2B).

Furthermore, the non-significant interaction effect (*F*_(1,20)_ = 0.14; *p* = 0.7167), and significant main effects of both duration (*F*_(1,20)_ = 13.12; *p* = 0.0017) and treatment (*F*_(1,20)_ = 5.43; *p* = 0.0303) were noted for the number of IBA1^+^ microglia in the hippocampal CA2 region. Both short-term control and EE mice showed significantly higher IBA1^+^ cell numbers than their respective long-term cohorts (43.8 ± 2.6 vs. 36.4 ± 2.6; *p* = 0.0323 and 50.0 ± 2.3 vs. 40.9 ± 1.3; p = 0.0210, respectively; Figure 2C).

For the number of IBA1^+^ microglia in the hippocampal CA3 region, the interaction effect was non-significant (*F*_(1,20)_ = 0.46; *p* = 0.5049) but the main effects of both duration (*F*_(1,20)_ = 42.64; *p* < 0.0001) and treatment (*F*_(1,20)_ = 25.30; *p* < 0.0001) were significant. Both short-term and long-term EE treated mice showed significantly higher IBA1^+^ cell numbers than their respective controls (74.2 ± 1.9 vs. 60.7 ± 3.0; *p* = 0.0013 and 57.1 ± 2.6 vs. 46.8 ± 1.8; *p* = 0.0119, respectively; Figure 2D). Similarly, both short-term control and EE mice showed significantly higher IBA1^+^ cell numbers than their respective long-term cohorts (60.7 ± 3.0 vs. 46.8 ± 1.8; *p* = 0.0005 and 74.2 ± 1.9 vs. 57.1 ± 2.6; *p* = 0.0001, respectively; Figure 2D).

#### Only Long-Term EE Induced a Significant Increase in the Number of GFAP^+^ Cells in the Dentate Gyrus and CA3 Regions of the Hippocampus

**Figure 3:**
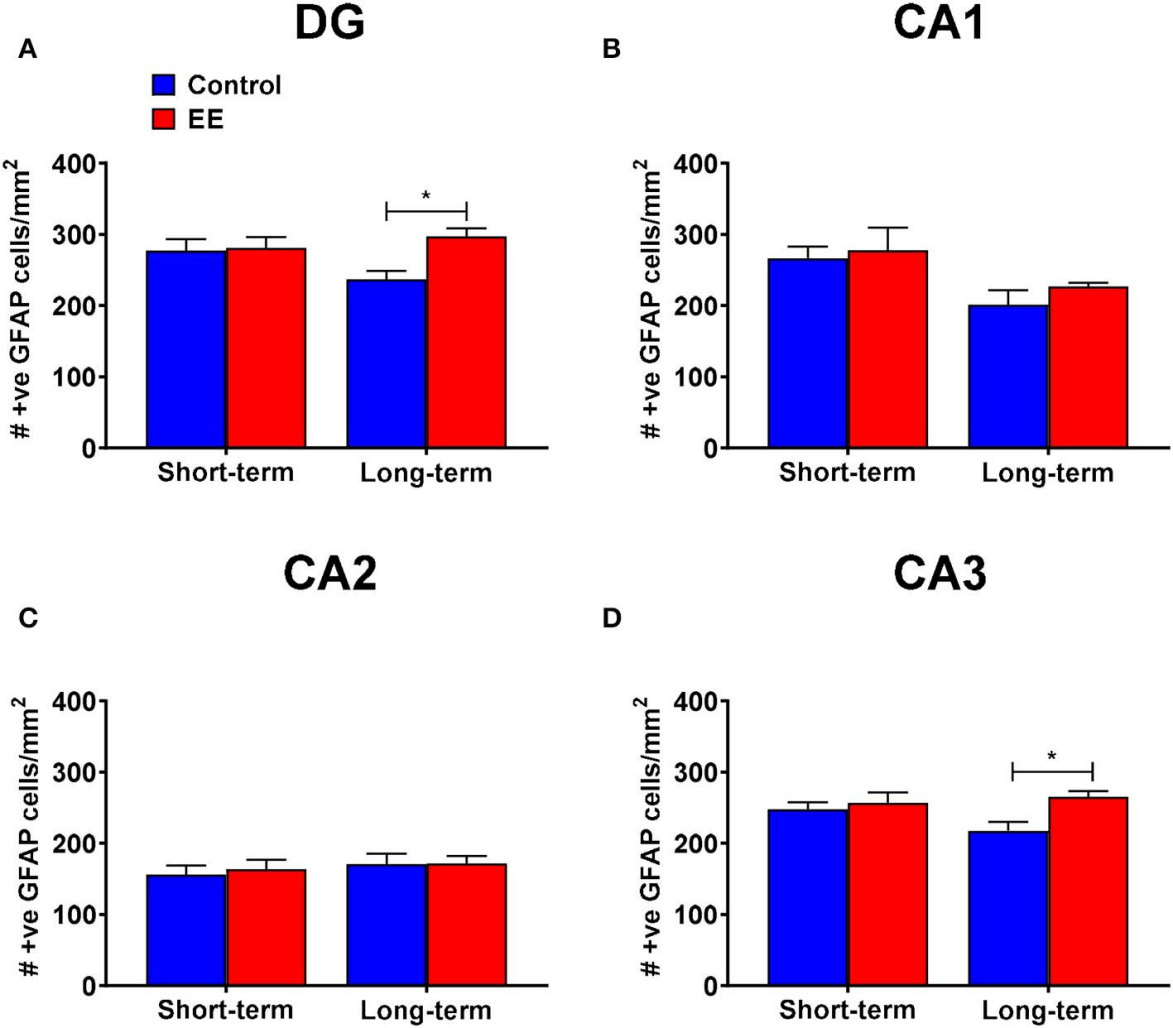
GFAP^+^ astrocytes in DG. **(A–D)** Number of GFAP^+^ astrocytes in the dentate gyrus (DG), CA1, CA2, and CA3 regions of the hippocampus, respectively. All data represented as mean ± SEM, *n* = 6 per group. *p < 0.05.

When we analyzed the number of GFAP^+^ astrocytes in the dentate gyrus, the significant interaction effect (*F*_(1,20)_ = 4.36; *p* = 0.0497) and the main effect of treatment (*F*_(1,20)_ = 5.56; *p* = 0.0286) were noted, but the main effect of duration (*F*_(1,20)_ =.83; *p* = 0.3739) was non-significant. On *post-hoc* analysis, long-term EE mice showed significantly greater number of GFAP^+^ cells than long-term controls (297.1 ± 11.3 vs. 236.6 ± 11.8; *p* = 0.0102; Figure 3A). Also, the number of GFAP^+^ astrocytes was low in long-term controls when compared to short-term controls at the significance level of *p* < 0.1 (*p* = 0.0912).

During the analysis of the number of GFAP^+^ astrocytes in the hippocampal CA1 region, we noted a non-significant interaction effect (*F*_(1,20)_ = 0.13; *p* = 0.7243) and the main effect of treatment (*F*_(1,20)_ = 0.81; *p* = 0.3777). The main effect of duration was significant (*F*_(1,20)_ = 8.02; *p* = 0.0103). On *post-hoc* analysis, no significant differences were observed between any of the groups (Figure 3B). However, the long-term controls showed lower number of GFAP^+^ astrocytes than short-term controls at the significance level of *p* < 0.1 (*p* = 0.0698).

The interaction effect (*F*_(1,20)_ = 0.07; *p* = 0.8002), as well as the main effects of both duration (*F*_(1,20)_ = 0.80; *p* = 0.3805) and treatment (*F*_(1,20)_ = 0.11; *p* = 0.7397) were non-significant for the number of GFAP^+^ astrocytes in the hippocampal CA2 region (Figure 3C).

For the number of GFAP^+^ astrocytes in the hippocampal CA3 region, both the interaction effect (*F*_(1,20)_ = 2.8; *p* = 0.1081) and the main effect of duration (*F*_(1,20)_ = 0.89; *p* = 0.3573) were non-significant, however, the main effect of treatment was significant (*F*_(1,20)_ = 6.15; *p* = 0.0221). *Post-hoc* analysis revealed significantly higher number of GFAP^+^ astrocytes in the hippocampal CA3 region of long-term EE mice when compared to long-term controls (265.5 ± 8.0 vs. 217.7 ± 12.7; *p* = 0.0160; Figure 3D).

### T Cell Immunophenotyping of Cervical Lymph Node Cells Exposed to Either Short or Long-Term EE

CD4+ (T helper) and CD8+ (cytotoxic T) cell proportions were measured as an indication of cellular immune response to the duration of the EE treatment. Representative images obtained during the FACS data analysis using FCS Express software are shown in Supplementary Figure 9.

**Figure 4:**
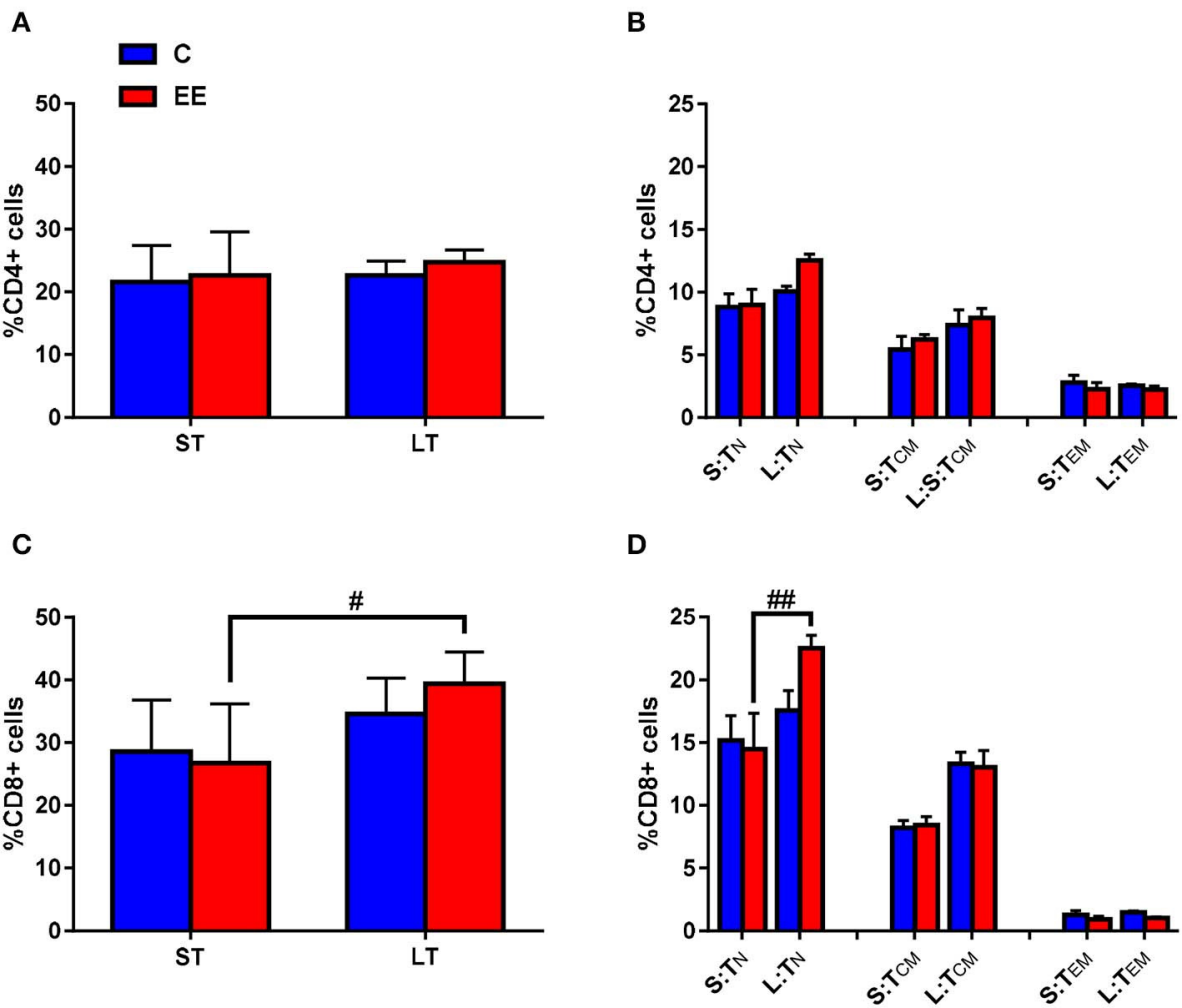
T-cell subset composition. Proportions of gated **(A)** CD4^+^ and **(C)** CD8^+^ T cells, and **(B)** naive, central memory and effector/effector memory CD4^+^ T cells and **(D)** naive, central memory and effector/effector memory CD8^+^ T cells in the cervical lymph nodes. All data represented in the proportion of CD4^+^ or CD8^+^ T cells as mean ± SEM, *n* = 6–8 per group. ^#^*p* < 0.05, ^##^*p* < 0.01. S, short-term; L, long-term; N, naïve; CM, central memory; EM, effector memory.

The change in the proportion of the gated CD4^+^ T cells was found to be non-significant in both short-term and long-term EE groups when compared to respective controls, with *p* > 0.05 for the interaction effect, as well as for the main effects of treatment and duration (Figure 4A). Likewise, no significant interaction effect and the main effects of the treatment and duration were observed for T_N_, T_CM_, and T_EM_ CD4^+^ T cells (*p* > 0.05; Figure 4B). However, two-way ANOVA, while still non-significant for the interaction effect and the main effect of treatment (p > 0.05), revealed significant main effect of the duration for the proportion of CD8^+^ T cell (*F*_(1,24)_ = 11.78; *p* = 0.0022). The proportion of CD8+ T cells was significantly higher in the long-term EE group when compared with the short-term EE group (39.4 ± 1.9 vs. 26.8 ± 3.8; *p* = 0.0195; Figure 4C). This was, in turn, the result of the long-term EE group showing a significantly higher proportion of the gated CD8^+^ T_N_ cell than the short-term EE group (22.5 ± 1.0 vs. 14.5 ± 2.9; *p* = 0.0016; Figure 4D).

The proportions of the T cell early activation marker CD25 on both CD4^+^ and CD8^+^ T cells were found to be non-significantly different between either short-term or long-term EE groups when compared to respective controls (*p* > 0.05, data not shown).

## Discussion

The objective of this study was to determine if the functional effects that we have reported in the recently published study (Singhal et al., 2019) were associated with changes in brain glial cells number and peripheral T cell subset proportion. Hence, we investigated the effect of duration of EE on glial cell (microglia and astrocytes) numbers in the hippocampus, and T cell subsets and phenotype in the cervical lymph nodes of mice that underwent behavioral battery. Since the distribution of microglia and astrocyte is not homogeneous between the four hippocampal regions (i.e., dentate gyrus, CA1, CA2, CA3) and may exhibit differences in glial cells number due to EE, we analyzed cells in all the four areas. However, there are a good number of evidence to suggest that EE alter the functions of the dentate gyrus region of the hippocampus, which regulates key brain functions, such as learning and memory (Nilsson et al., 1999; Kim et al., 2004; Bruel-Jungerman et al., 2005; Chen et al., 2006; Williamson et al., 2012). EE, regardless of duration, increased the number of IBA^+^ microglia within the dentate gyrus, CA1 and CA3 regions of the hippocampus. Conversely, only the long-term EE enhanced GFAP^+^ astrocyte number within the dentate gyrus and CA3 regions of the hippocampus when compared to control mice. Evaluation of the T cell population within the cervical lymph nodes revealed that both short-term and long-term EE did not affect the proportion of CD4^+^ T cells and CD4^+^ T cell subsets. However, long-term EE mice showed a significantly higher proportion of CD8^+^ T cells compared to short-term mice, which we observed was due to the significantly higher proportion of CD8^+^ T_N_ cells in long-term EE mice. Both short-term and long-term EE showed no effect on the proportion of T cell early activation marker CD25.

### Duration of EE Affects Astrocyte but Not Microglial Numbers in the Dentate Gyrus at Middle Age

Astrocytes and microglia are the primary immune effector cells in the CNS and regulate the extensive bi-directional communication between the nervous and the immune systems in response to different immunological, physiological and psychological stressors, as well as external laboratory treatments. Indeed, in constitutive states, they are essential for the maintenance of neurobiological homeostasis and play a vital role in neuroprotection and neuroplasticity (Hamm et al., 1996; Passineau et al., 2001). Microglia are important for tissue maintenance and repair and maintaining homeostasis in the CNS (Michell-Robinson et al., 2015), whilst astrocytes provide a structural framework to the blood-brain-barrier, regulate the transmission of electrical impulses within the brain, release glycogen and provide nutrients, such as lactate, to the nervous tissue, maintain extracellular ion balance, and repair damaged tissues within the brain (Walz, 2000; Parri and Crunelli, 2003; Figley and Stroman, 2011; Anderson et al., 2016). Glial cells are not only altered in density and function during psychiatric disorders but also under improved environmental conditions, particularly in the hippocampus. As such, measuring the change in the number of the astrocytes and microglia in the dentate gyrus, the region widely associated with altered cognitive function and affect regulation, can provide satisfactory evidence pointing toward an association of EE on neuroimmune homeostasis. It must be noted that during old age, the dysregulated functioning of microglia and astrocytes leads to enhanced glial pro-inflammatory cytokine signaling, impaired neurobiology, and an increase in apoptotic cell death (Kuzumaki et al., 2010).

We investigated the effects of EE in middle-age mice that were sacrificed immediately after the behavioral testing. Since mice did not grow old and were housed in a controlled environment devoid of external stressors, we hypothesized an increase in the number of both immunopositive microglia and astrocyte with enhanced productive functions after EE. We indeed observed an increase in microglial numbers within the dentate gyrus, CA1, and CA3 regions after both short- and long-term EE. The increase in the number of IBA^+^ microglia in the dentate gyrus after EE is consistent with the previously published findings in 2 months old male Sprague-Dawley rats, however, unlike our results, the authors reported no change in the immunopositive microglia number within the CA1 and CA3 regions of the hippocampus (Williamson et al., 2012). An increase in the number of microglia in the dentate gyrus at middle age after EE indicates enhanced neuroprotection. In our published behavioral study, we have reported enhanced baseline locomotion and reduced depressive-like behavior after both short and long-term EE (Singhal et al., 2019). Taken together, we believe the reported improved behavioral outcomes after the EE could be associated with the increased number of microglia in the hippocampus, in particular within the dentate gyrus of mice. Interestingly, we also observed significantly higher IBA1^+^ cell numbers in the CA1, CA2, and CA3 regions of short-term control and EE mice when compared to their respective long-term cohorts. However, since the differences were observed both in control and EE mice, we attribute this to the experimental variability and not to the effects of duration of EE or the starting age of EE treatment.

We observed no effects on the number of GFAP^+^ astrocytes in any of the hippocampal regions after the short-term EE, but the number of GFAP^+^ astrocytes increased significantly after the long-term EE in the dentate gyrus and CA3 regions of the hippocampus. It is, therefore, likely that astrocytes are less reactive to EE than microglia and a longer duration of EE may be required to significantly alter astrocytes number in the brain. Our results contradict the previously reported findings of a significant increase in the number of astrocytes in 2-months-old C57BL/6 female mice after 7 weeks of EE, although the increase was shown in layer 1 of the motor cortex and not in the hippocampus (Ehninger and Kempermann, 2003). Similarly, another study reported a significant increase in the number of GFAP^+^ astrocyte within the dentate gyrus, but not in the CA3 region, of 2-months-old male Sprague-Dawley rats, housed for 12 h in an enriched environment daily for seven weeks (Williamson et al., 2012). This possibly suggests that astrocytes are more reactive to EE at an early age but not at middle age. We believe ours is the first report of an increase in the number of GFAP^+^ astrocytes in the dentate gyrus after long-term EE at middle age. Long-term EE of 3 months, from 5–8 months of age, in the presence of a running wheel, has also been shown to increase the number of astrocytes in a mouse model of Alzheimer's disease (Beauquis et al., 2013). However, it must be noted that physical exercise alone can enhance the number of hippocampal GFAP^+^ astrocytes (Saur et al., 2014). The evidence mentioned above suggests that the age of the rodents, duration of EE, as well as the presence or absence of running wheels, may alter the effects of the duration of EE on astrocyte numbers within the hippocampus.

Interestingly, an increase in both microglia and astrocytes number in the dentate gyrus of the middle age long-term EE mice was also accompanied by an increase in anxiety-like behavior that we have reported in the recently published study (Singhal et al., 2019). We observed that this could be because of reduced activity of long-term EE mice in the challenging conditions of brightly lit open field and elevated zero maze after 5 months of habituation to an enriched environment in basal home conditions. Indeed, long-term EE mice showed a significant reduction in locomotion in the stressful environment of Open Field. We, therefore, believe that the increased anxiety of long-term EE mice may not necessarily be associated with an increase in microglia or astrocytes number in the dentate gyrus that we have reported in this study.

### Only Long-Term EE Alters Peripheral T Cell Proportion at Middle Age

Ours is the first study to investigate the change in the proportion of T cell subsets in the cervical lymph nodes in response to different durations of EE. The analysis of the total T cell counts, including CD4^+^ (helper T cells) and CD8^+^ (cytotoxic T cell), helped us to determine the cellular immune response after short-term and long-term EE treatments. We observed that altered CD8^+^ (cytotoxic T) but not CD4^+^ (T helper) cell proportions were associated with the duration of the EE treatment.

Mature T_N_ cells proliferate and convert into T_CM_ or T_EM_ cells on the recognition of a cognate antigen. T_CM_ cells have little or no effector function but mediate reactive memory—proliferate and differentiate to effector cells in response to antigenic stimulation. T_EM_ cells, which are short-lived, migrate to the site of infection, eliminate the antigenic molecular patterns and undergo apoptosis once the antigens are eliminated. However, some T_EM_ cells also differentiate into memory cells that provide long-term adaptive immunity. Our results suggest that the duration of EE has no differential effects on the proportion of CD4^+^ T cells and their subsets. However, a longer duration of EE may be beneficial by supporting an increase in the proportion of cytotoxic CD8+ T and CD8^+^ T_N_ cells as found in middle age when compared with those with short term EE.

T_N_ cells also respond by acquiring activated phenotypes and upregulation of early T cell activation markers, such as CD25, which are important in the maintenance of tolerance to self-antigen and prevention of autoimmune disorders (Simms and Ellis, 1996; Rosenkranz et al., 2007; Ha, 2009). However, no differences in CD4^+^ and CD8^+^ early activation T cell markers were observed after both short- and long-term EE. Our results are in contrast to the known neuroprotective effects of EE (Young et al., 1999; Singhal et al., 2014a; Bardi et al., 2016). Nonetheless, the enhanced cytotoxic immunity could be attributed to the observed decrease in locomotion in a threatening environment (open field) and increases in anxiety-like behavior in middle-aged C57BL/6 mice after long-term EE that we have reported in our recent publication (Singhal et al., 2019).

The controlled environment, absence of pathogens and stressful stimuli, age and strain of the rodents, EE paradigm structure, size and traits of EE objects, and the frequency of changing the objects that we utilized during our study, may all have a role to play in the observed findings. Further research into the differential effect of the various EE objects on neuroimmune functions in older mice may be required. Furthermore, the results need to be followed up in old-age and psychiatric disease rodent models to further validate and extend the findings. Additionally, since running wheels have been used as an EE tool in past studies, the differential effects of long-term EE, long-term PE, and their combination, need to be explored.

### Limitations of the Study

Our study is the first to investigate the effects of EE on T cells and T cell subset proportions from the cervical lymph nodes. Furthermore, we conducted behavioral testing for 3 weeks on mice immediately before molecular analysis. Hence external factors, such as social or handling stress (Hardy et al., 1990; Stefanski and Engler, 1999; Stefanski, 2001) and sensory stimulation (Roberts, 1995, 2000) during behavioral testing, could have modulated the brain glia number and cervical lymph node T cell subset proportion. However, it must be noted that all mice underwent the same behavioral testing protocol which should control for any of these effects. It is also possible that the immune response may not have been strong in response to treatments at middle age (9-months) in C57BL/6 mice.

Furthermore, it is the primed phenotype of glial cells that drives functional changes. This encompasses much more than an increase in the number of immunopositive glial cells in the dentate gyrus. Hence, examining the morphology of glial cells and levels of other activation markers, for example, cytokines, chemokines, C-reactive protein, and protein kinases are required to develop a full understanding of neuroimmune response after the short- and long-term EE treatments. Our results, therefore, do not make a full profile of the neuroimmune response and may not fully explain the reported change in brain functions after the two treatments, however, we believe these provide a head start and would contribute significantly to the understanding of overall neuroimmune changes in response to EE together with future work.

Another limitation of our study is that we could not analyze the effect of sex due to the low sample size (when the groups were divided into males and females). Sex could have significant effects on the reported results and as such we believe an independent study investigating a three-way interaction between treatment, duration of EE and sex would be very useful. Also, although the starting age of long-term EE mice is different from short-term EE mice, it was not possible to have the same starting age. In the latter case, we would have been comparing mice of different ages during behavioral testing (after 4 weeks and 6 months of EE). This would have led to even greater differences not related to the EE since aging is a known risk factor for change in brain functions and underlying molecular factors. The questions we have raised should be explored in future EE studies on rodents, including those in preclinical models of brain disorders.

## Conclusion

Our EE paradigm differed from the many previous published EE protocols in not having running wheels. We focused on stimulating the environment with toys, tunnels, ladders, and other accessories with extra bedding to stimulate cognitive areas in the absence of wheel-running physical activity. Overall, our results suggest that EE with stimulating cognitive objects for both short-term and long-term increases in the number of immunoreactive microglia, but long-term EE is required to modulate the number of immunoreactive astrocytes, and peripheral T lymphocyte proportion and phenotype during middle age in C57BL/6 mice. Together with the results from our published behavioral study (Singhal et al., 2019), it is clear that that the duration of EE has subtle effects on behaviors and may also determine the astrocytic and peripheral T lymphocytes' response.

## Data Availability Statement

The datasets generated for this study are available on request to the corresponding author.

## Ethics Statement

The animal study was reviewed and approved by University of Adelaide Animal Ethics Committee. Ethics approval number M216-12.

## Author Contributions

Material preparation, data collection, and analysis were performed by GS. JMo assisted GS with the mouse husbandry work and collection of data. FC, CT, and JMa assisted GS with the molecular analysis. AH provided technical guidance during the study as and when needed. FC, MJ, EJ, and BB supervised the project. The first draft of the manuscript was written by GS. All authors commented on previous versions of the manuscript, read and approved the final manuscript and contributed to the study conception and design.

### Conflict of Interest

The authors declare that the research was conducted in the absence of any commercial or financial relationships that could be construed as a potential conflict of interest.
