# Supplementary material for: Duration of Environmental Enrichment Determines Astrocyte Number and Cervical Lymph Node T Lymphocyte Proportions but Not the Microglial Number in Middle-Aged C57BL/6 Mice
Source: Front Cell Neurosci. 2020 Mar 18;14:57. doi: 10.3389/fncel.2020.00057 (PMC7094170; doi:10.3389/fncel.2020.00057)
Supplement: Supplementary file 1 [file Data_Sheet_1.docx]

**Supplementary Figures**

**
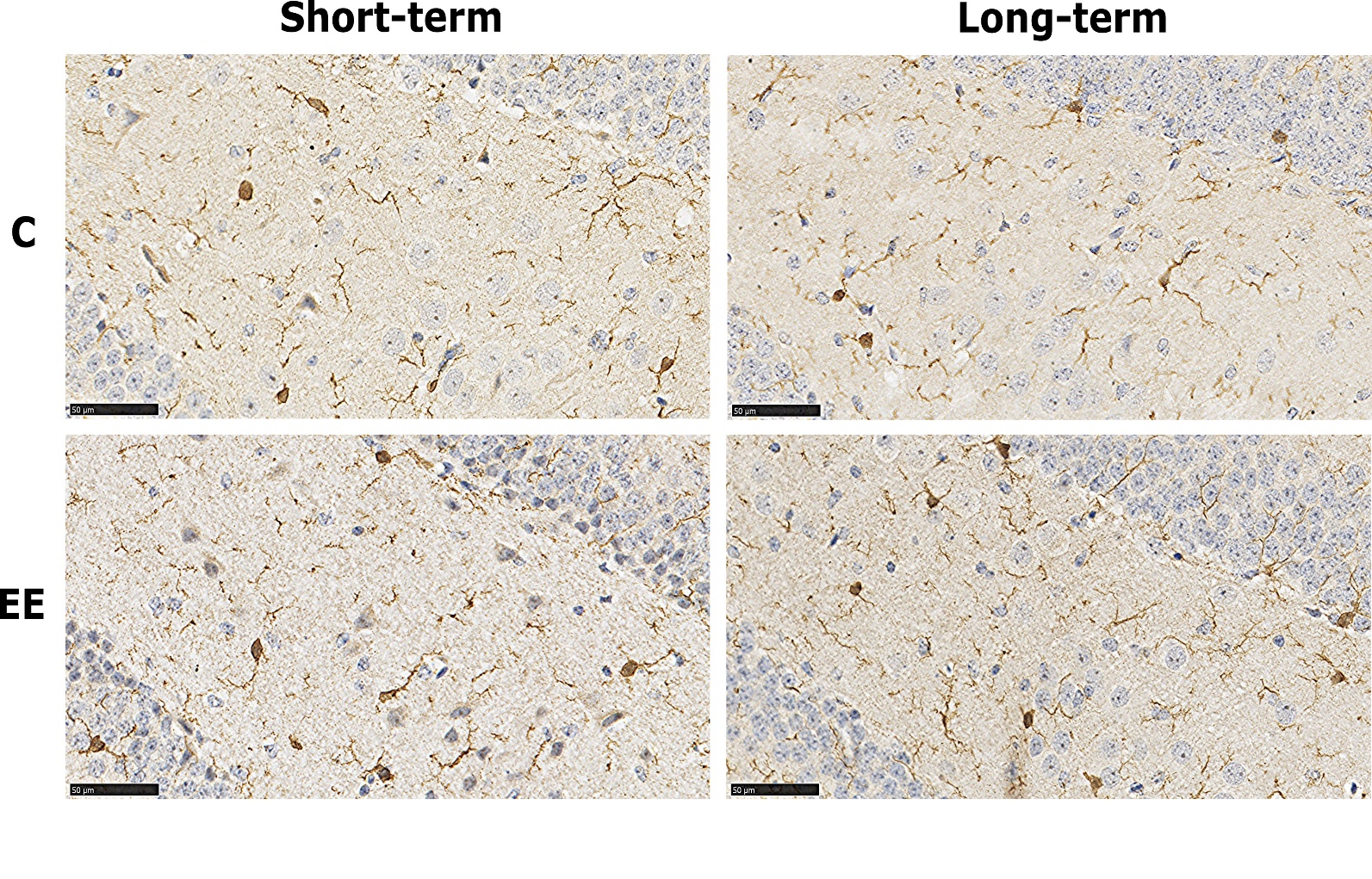
**

**S. Fig. 1.** Representative immunohistochemical images of the number of IBA1+ microglia in the dentate gyrus region of the hippocampus in short- and long-term control and EE groups (the black scale represents 50µm length).


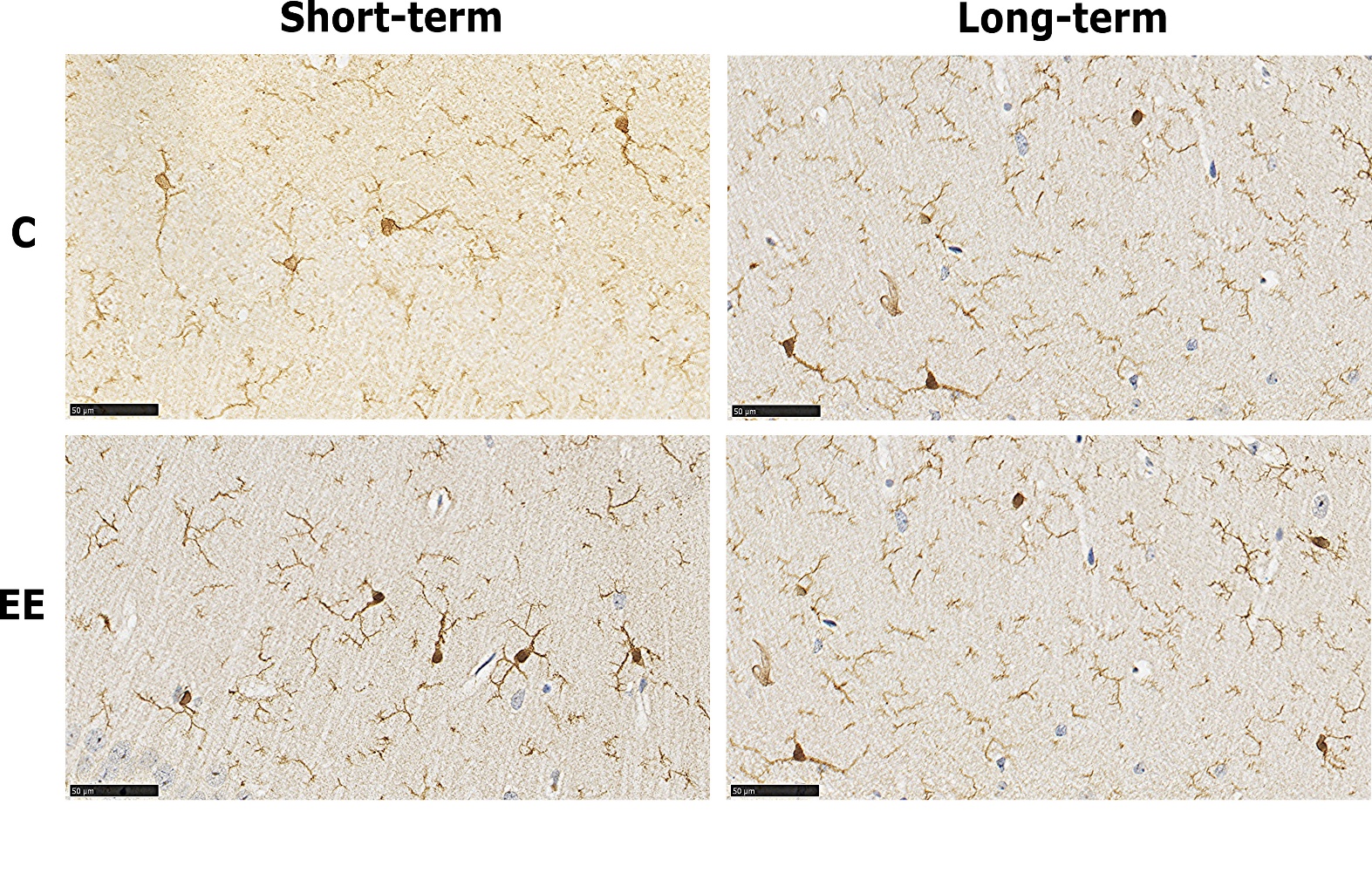
**S. S. Fig. 2.** Representative immunohistochemical images of the number of IBA1+ microglia in the CA1 region of the hippocampus in short- and long-term control and EE groups (the black scale represents 50µm length).


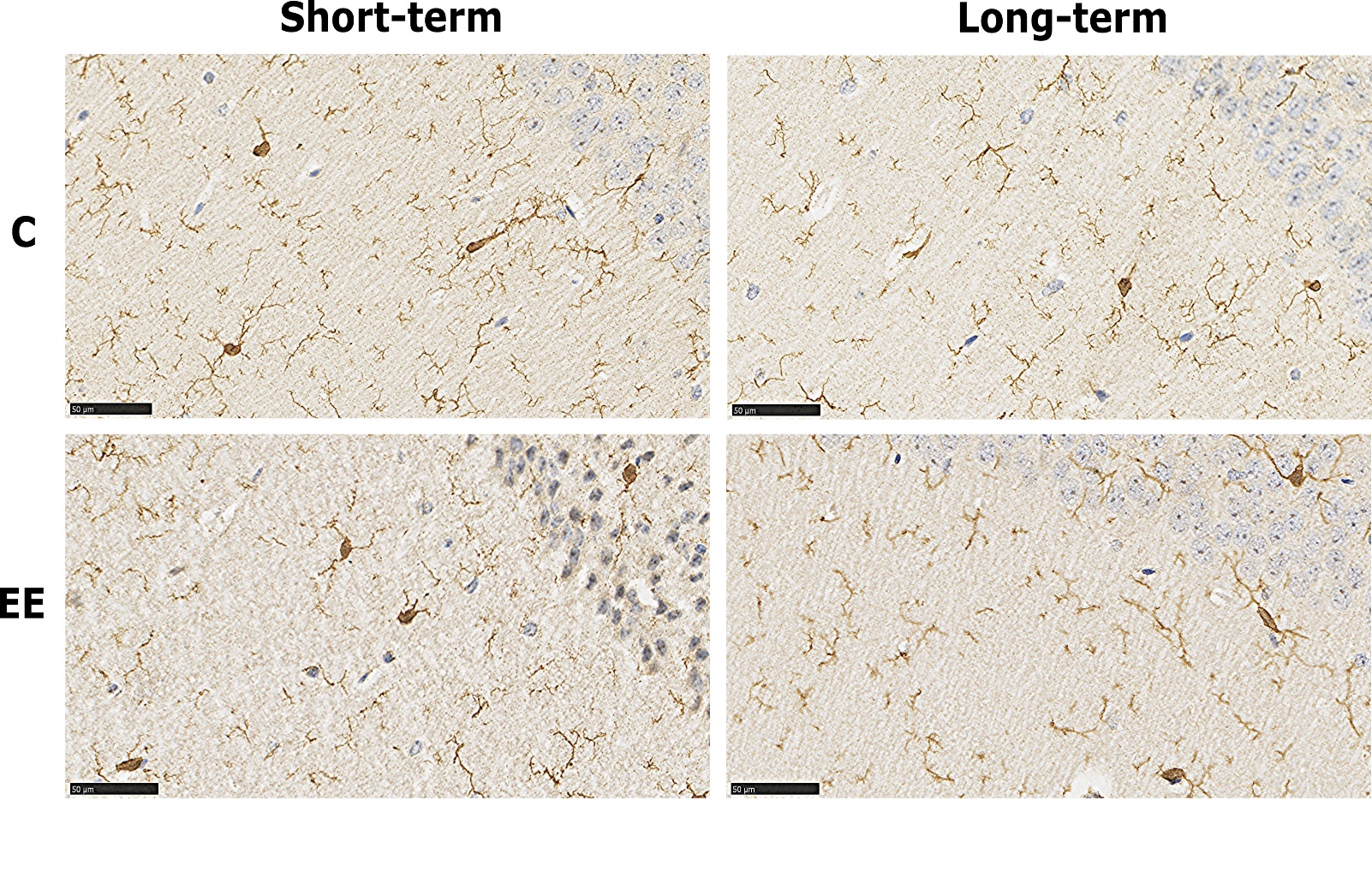


**S. Fig. 3.** Representative immunohistochemical images of the number of IBA1+ microglia in the CA2 region of the hippocampus in short- and long-term control and EE groups (the black scale represents 50µm length).


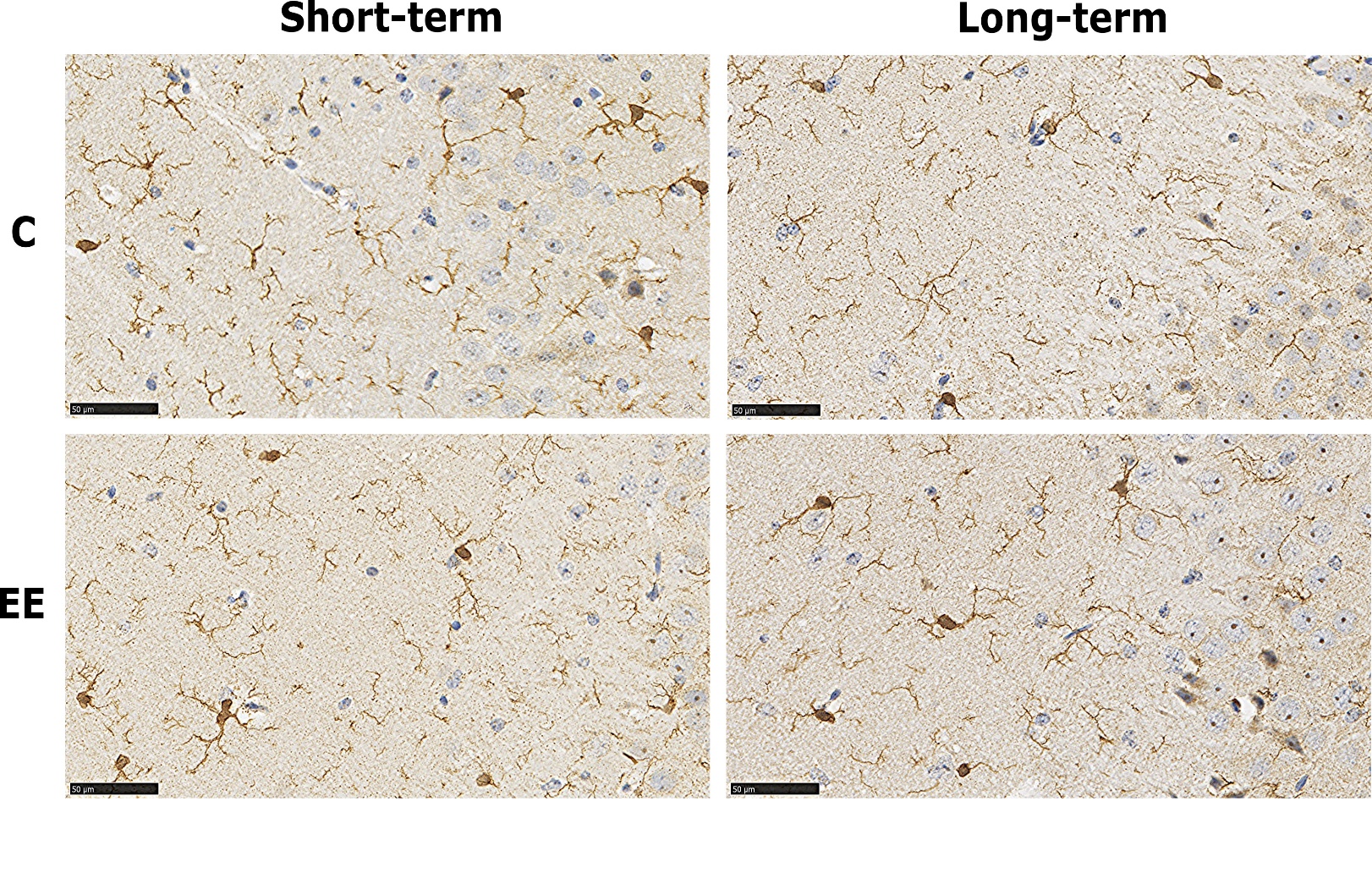


**S. Fig. 4.** Representative immunohistochemical images of the number of IBA1+ microglia in the CA3 region of the hippocampus in short- and long-term control and EE groups (the black scale represents 50µm length).

**
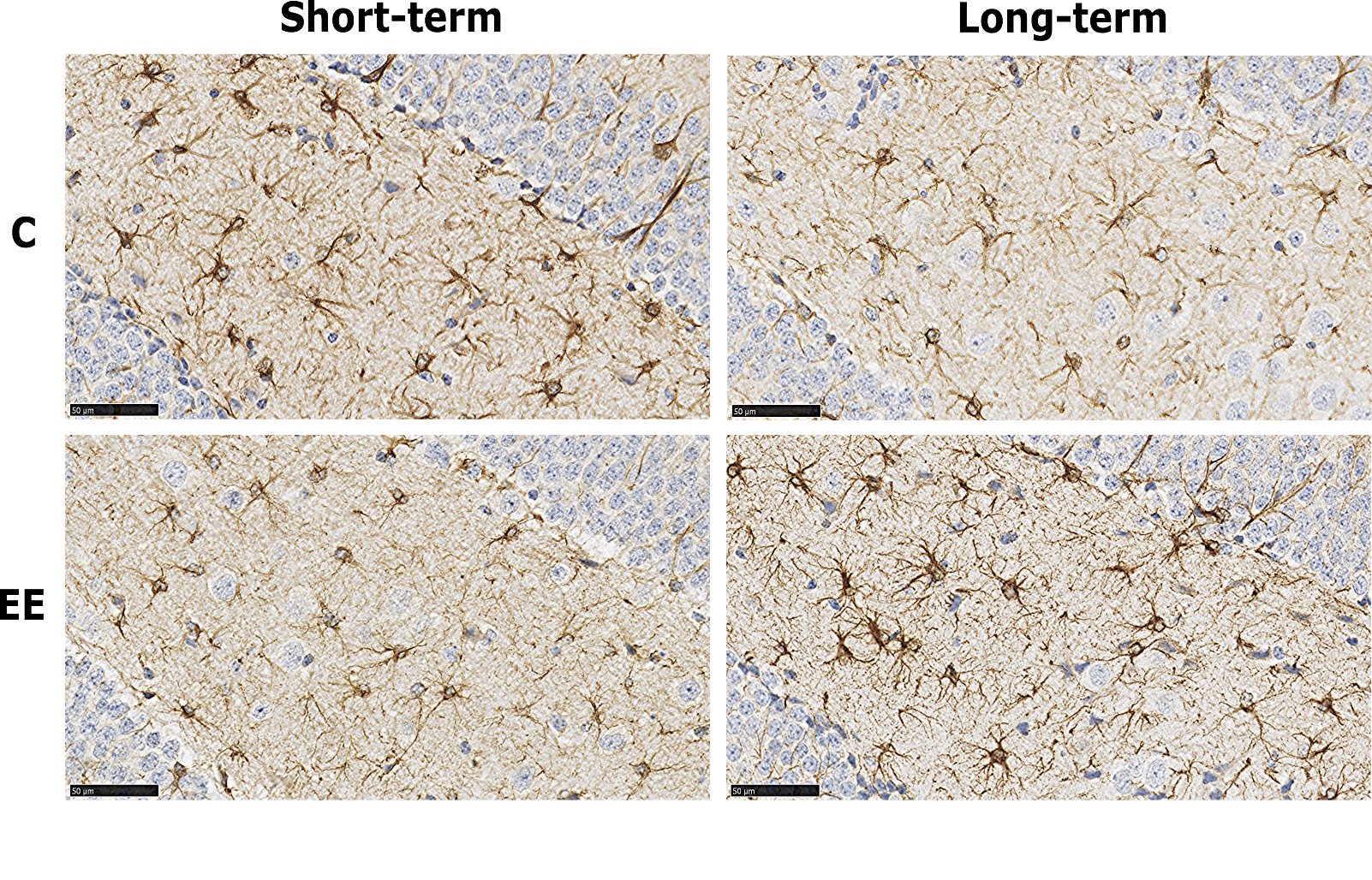
**

**S. Fig. 5.** Representative immunohistochemical images of the number of GFAP+ astrocytes in the dentate gyrus region of the hippocampus in short- and long-term control and EE groups (the black scale represents 50µm length).


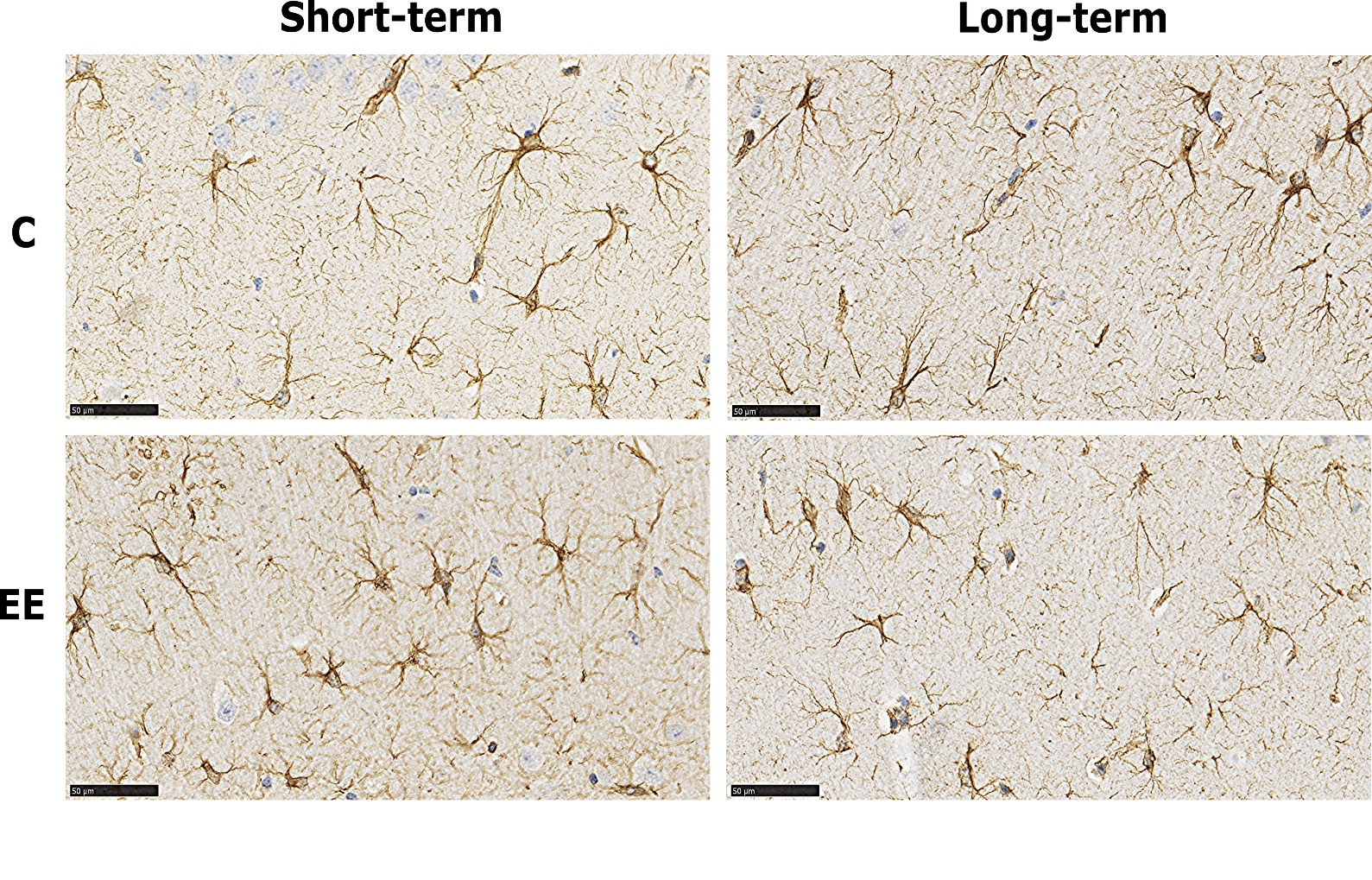


**S. Fig. 6.** Representative immunohistochemical images of the number of GFAP+ astrocytes in the CA1 region of the hippocampus in short- and long-term control and EE groups (the black scale represents 50µm length).

**
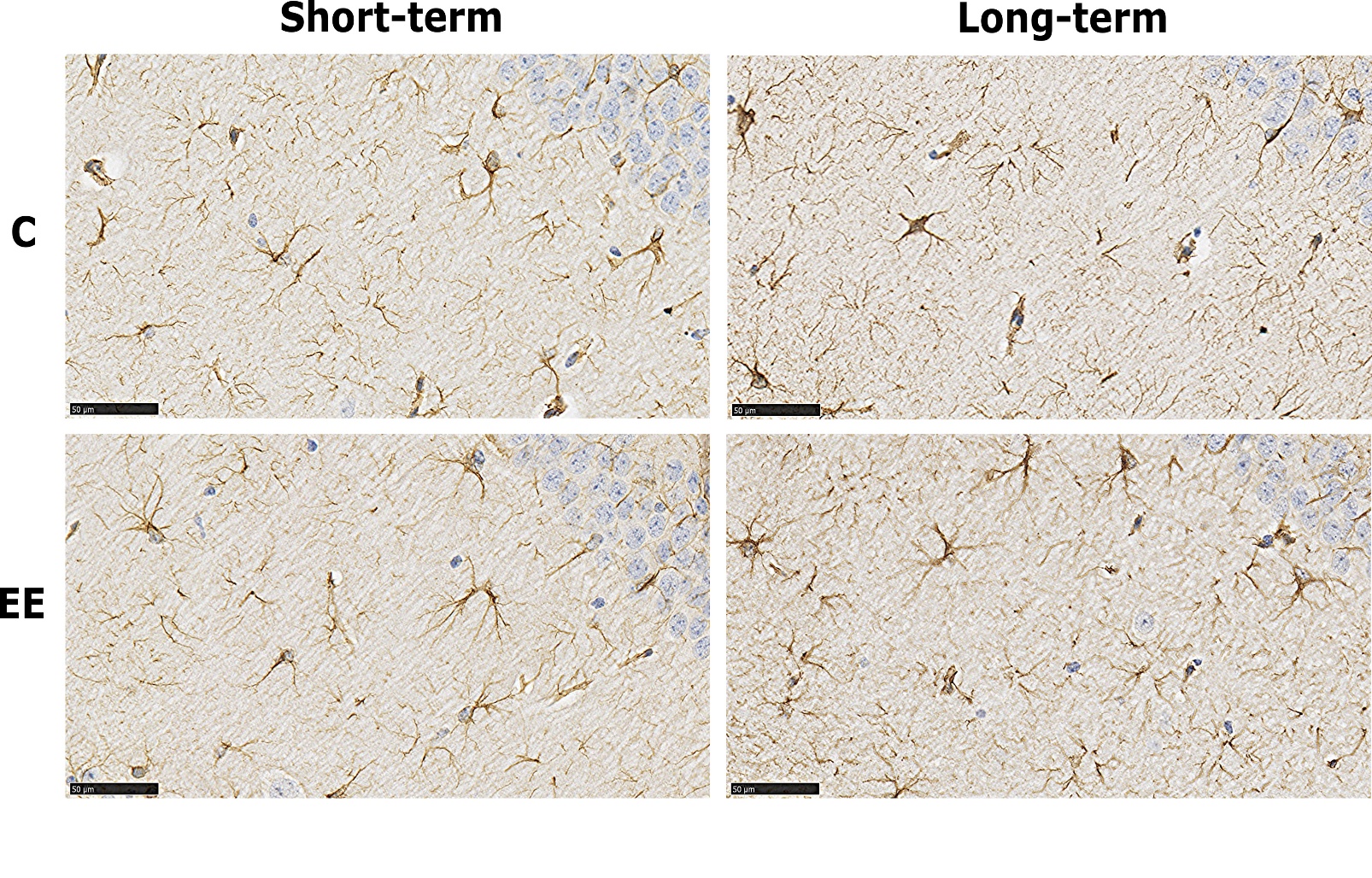
**

**S. Fig. 7.** Representative immunohistochemical images of the number of GFAP+ astrocytes in the CA2 region of the hippocampus in short- and long-term control and EE groups (the black scale represents 50µm length).

**
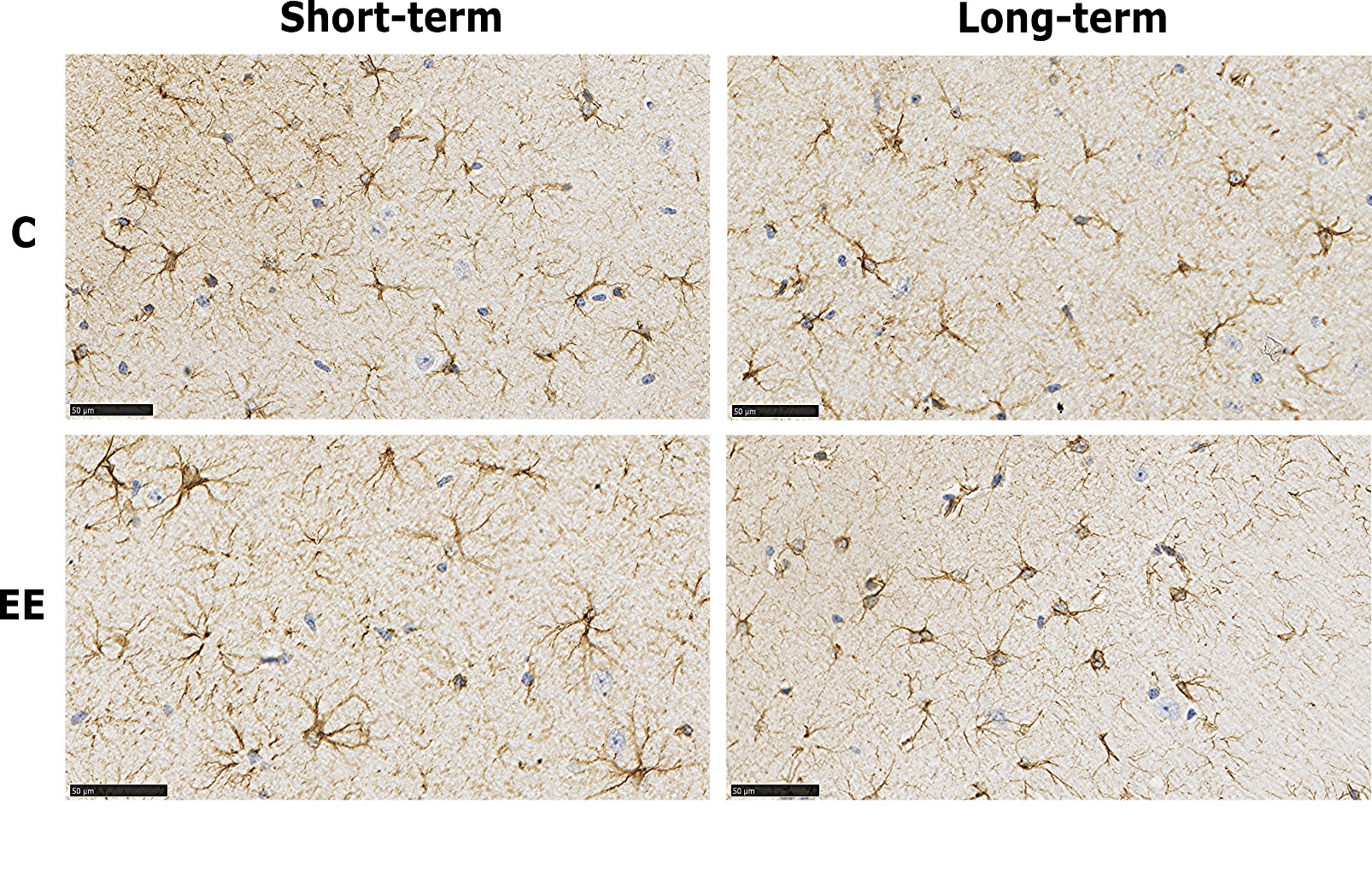
**

**S. Fig. 8.** Representative immunohistochemical images of the number of GFAP+ astrocytes in the CA3 region of the hippocampus in short- and long-term control and EE groups (the black scale represents 50µm length).

**
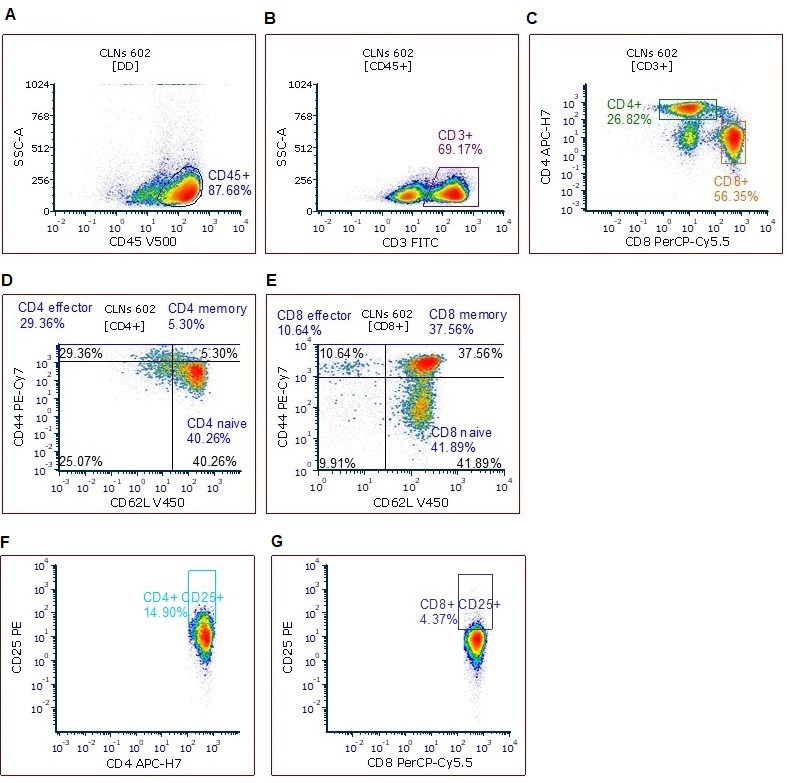
**

**S. Fig. 9.** A-E: Representative of the density plots showing (A) Gated CD45^+^ cells, (B) CD3^+^ cells derived from gated CD45+ cells, and (C) CD3^+^ CD4^+^ and CD3^+^ CD8^+^ T cells distinguished from total CD3^+^ gated cells. Further gating on CD44^+^ and CD62L^+^ cell populations enabled the identification and estimation of (D) CD4^+^ and (E) CD8^+^ T cell subsets, i.e., Naïve (T_N_), Central memory (T_CM_) and Effector memory (T_EM_) T cells. F-G: Representative of the density plots showing the proportion of early activation markers CD25^+^ on (F) CD4+ and (G) CD8+ T cell subpopulations derived from the gated CD45+ T cells.
